# Safety and Transfer Study: Transfer of Bromoform Present in *Asparagopsis taxiformis* to Milk and Urine of Lactating Dairy Cows

**DOI:** 10.3390/foods10030584

**Published:** 2021-03-10

**Authors:** Wouter Muizelaar, Maria Groot, Gert van Duinkerken, Ruud Peters, Jan Dijkstra

**Affiliations:** 1 Wageningen Livestock Research, Wageningen University & Research, P.O. Box 338, 6700 AH Wageningen, The Netherlands; gert.vanduinkerken@wur.nl; 2 Animal Nutrition Group, Wageningen University & Research, P.O. Box 338, 6700 AH Wageningen, The Netherlands; jan.dijkstra@wur.nl; 3 Wageningen Food Safety Research, Wageningen University & Research, P.O. Box 230, 6700 AE Wageningen, The Netherlands; maria.groot@wur.nl (M.G.); ruudj.peters@wur.nl (R.P.)

**Keywords:** safety, transfer, cattle, seaweed, CHBr_3_, rumen, *Asparagopsis*, toxicology

## Abstract

Enteric methane (CH_4_) is the main source of greenhouse gas emissions from ruminants. The red seaweeds *Asparagopsis taxiformis* (AT) and *Asparagopsis armata* contain halogenated compounds, including bromoform (CHBr_3_), which may strongly decrease enteric CH_4_ emissions. Bromoform is known to have several toxicological effects in rats and mice and is quickly excreted by the animals. This study investigated the transfer of CHBr_3_ present in AT to milk, urine, feces, and animal tissue when incorporated in the diet of dairy cows. Twelve lactating Holstein-Friesian dairy cows were randomly assigned to three treatment groups, representing the target dose (low), 2× target dose (medium), and 5× target dose (high). The adaptation period lasted seven days, and subsequently cows were fed AT for 22 days maximally. The transfer of CHBr_3_ to the urine at days 1 and 10 (10–148 µg/L) was found with all treatments. On day 1, CHBr_3_ was detected in the milk of most cows in the low and medium treatment groups (9.1 and 11 µg/L, respectively), and detected in the milk of one cow in the high treatment group on day 9 (35 µg/L). Bromoform was not detected in milk and urine at day 17, nor at concentrations above the detection limit in feces and collected animal tissues. Two animals (low) were sacrificed, and their rumen wall showed abnormalities. Upon histological examination, signs of inflammation became visible. Animals regularly refused the feed or distinctively selected against AT. In conclusion, within the confines of the present experiment, CHBr_3_ does not accumulate in animal tissue, but can be excreted in urine and milk.

## 1. Introduction

Enteric methane (CH_4_) production is a main target of greenhouse gas mitigation practices in livestock, and several mitigation strategies have been proposed [1]. Halogenated methane analogues, including bromoform (CHBr_3_), have been shown to inhibit CH_4_ formation in the rumen of sheep [2]. In recent years, the use of CHBr_3_ containing seaweeds (i.e., *Asparagopsis taxiformis* and *Asparagopsis armata*) to mitigate enteric CH_4_ emission of cows has gained increasing attention. The red seaweed *A. taxiformis* effectively reduces CH_4_ production in in vitro gas production systems [3,4,5], with 99% reduction in CH_4_ production, and an inclusion rate of 2% organic matter (OM) basis [6]. The most likely bioactive compound that is responsible for the observed reductions in CH_4_ emissions and present in sufficient quantities in *A. taxiformis* was CHBr_3_ [7]. The underlying mechanism might be related to the reduced efficiency of the cobamide-dependent methyl transferase by interacting with reduced vitamin B_12_ [8], a crucial step of methanogenesis in the rumen.

In vivo research with sheep (n = 29, two years old) showed an 80% reduction in CH_4_ emission by *A. taxiformis* (1.75 mg/kg dry matter (DM) CHBr_3_), as mentioned by Kinley et al. [9], with an inclusion rate of 3% on an OM basis [10]. In the same study, four animals (one control, three treatments) were pre-emptively removed before CH_4_ measurements due to decreased dry matter intake (DMI) over three consecutive days, and eleven animals regularly refused the offered *A. taxiformis*. No effects on DMI were apparent in this study, because animals were fed restrictively (1.2× maintenance), and several animals did not consume all of the *A. taxiformis* [10]. During necropsy, five of the 12 examined sheep, all in *A. taxiformis* groups, showed pathological changes of the ruminal mucosa, consisting of granulomatous (i.e., collection of macrophages) and keratotic changes (i.e., overgrowth of keratinocytes, macroscopically leading to whitish tan discoloration and blunting of papillae) [10]. Incorporating *A. armata* (1.32 mg of CHBr_3_/g DM) in the diet of dairy cows reduced enteric CH_4_ yield by 20.5% and 42.6% for 0.5% and 1.0% OM inclusion, respectively [11]. The DMI decreased significantly by 10.8 and 38.0%, with an 0.5 and 1.0% OM inclusion of *A. armata* [11]. In the control group, low levels of CHBr_3_ in the milk (0.11 µg/L) were found, and did not increase significantly in the treatment groups by inclusion of *A. armata* in the diet (0.15 µg/L, both treatments) [11]. Incorporating *A. taxiformis* (6.55 mg/g DM) in the diet of beef steers at 0.1% and 0.2% OM inclusion led to a 38% and 98% reduction in CH_4_ yield, respectively, with no significant reduction at 0.05% OM inclusion [9]. No CHBr_3_ residues were detected in any of the collected meat, kidney, fat, or feces samples [9]. The main focus of all experiments was to study the effect of Asparagopsis seaweed on CH_4_ emission by ruminants. The sheep and beef cow experiments also focused on the transfer of CHBr_3_ to animal tissues and effects on rumen health, while the study in dairy cows only examined the transfer to milk. No study in dairy cows has been performed to examine the CHBr_3_ residues in animal tissues, milk, urine, and feces and the effects on ruminal health.

Toxicological research with CHBr_3_ has only been performed with rats and mice. In rats, pure CHBr_3_ increased blood carbon monoxide concentration, leading to potentially hazardous levels of carboxyhaemoglobin [12], and administration of pure CHBr_3_ was linked to kidney and liver toxicity in male mice [13]. To the best of our knowledge, no data of toxicological research on CHBr_3_ are available for ruminants, and no toxicity data are available of CHBr_3_ when present in a carrier such as a seaweed, like *A. taxiformis*.

The aim of this study was therefore to investigate the possible transfer of CHBr_3_ from *A. taxiformis* to milk, urine, feces, and animal tissues when offered to dairy cows. Moreover, the transfer study was also used as a case study to gather more insights on the effects of feeding *A. taxiformis* on animal health, ruminal changes, feed intake, milk production, and milk composition.

## 2. Materials and Methods

The experiment was conducted from mid-August until mid-September 2019 at the animal research facilities of Wageningen University and Research (Leeuwarden, the Netherlands), lasted for four weeks, was in accordance with Dutch law, and approved by the Central Authority for Scientific Procedures on Animals (CCD, the Hague, the Netherlands; 2016.D-0108.012). The experiment was designed according to the principles of EFSA Guidance for establishing the safety of additives for the consumer following guidelines for a residue study [14].

### 2.1. Experimental Design, Diets, Feeding, and Housing

Throughout the experiment, all cows were housed in a tie stall with rubber mats covered with wood shavings as bedding material. All cows were able to see, hear, and smell each other, and lights inside the barn were on from 05:00 to 23:00 h. Feed was offered in individual feeding bins (Insentec, Hokofarm Group BV, Marknesse, the Netherlands), and cows had free access to clean drinking water throughout the experiment.

Twelve non-pregnant Holstein-Friesian dairy cows (seven primiparous, five multiparous), averaging 339 ± 135.7 days in milk (DIM, mean ± SD) and 664 kg ± 81.1 in bodyweight (BW), were randomly allocated to one of three dietary treatments at day −7 relative to the first offering of seaweed mix. The intended experimental design included 35 days, consisting of an adaptation period of seven days (no seaweed mix offered) and an experimental period of 28 days (seaweed mix offered). During the adaptation and experimental period, all animals received the same basal diet. Animals received a fixed amount of *A. taxiformis* per day. Treatments consisted of 67 g DM *A. taxiformis* (AT) (low), 133 g DM AT (medium) and 333 g DM AT (high) daily. The low treatment group consisted of eight animals (cows L1 until L8, 359 ± 161 DIM, 648 kg ± 84.2 BW), whereas medium (cows M1 and M2, 314 ± 34 DIM, 758 kg ± 1 BW) and high (cows H1 and H2, 282 ± 0.5 DIM, 627 kg ± 16 BW) treatment groups both consisted of two animals. Less animals were used in the medium and high treatments because these scenarios are less likely to occur in practice and induce higher risks for the animals. The low treatment was the target treatment, medium represented an accidental overdosing (2× low) by the farmer or animal (by overeating) that may occur in practice, and high was considered to represent a worst-case scenario (5× low). *Asparagopsis taxiformis* was harvested on 28 May 2019, at Salão, Faial, Azores, and stored at −25 °C until freeze drying between 3 and 5 June 2019 (European Freeze Dry, Kirke Hyllinge, Denmark), sealed in air-tight and light-reflecting bags, and stored at room temperature before shipping. Before the start of the experiment, a test with a limited amount of animals was carried out to determine the best method to offer *A. taxiformis*. It was determined that, for all treatments, *A. taxiformis* was offered in a mix of dextrose, wheat, dehydrated beet pulp, and water, called seaweed mix thereafter. The seaweed mix composition per treatment is presented in Table 1. All animals received the same basal diet consisting of grass silage, maize silage, compound feed (60% beet pulp, 40% wheat; DM basis), soybean meal, and wheat straw at the ratio of 57:25:12:3:2 (DM basis). With the aim to assure controlled maximum uptake, the seaweed mix was offered twice daily before the basal diet was offered. The animals had 1–2 h to finish the seaweed mix, the seaweed mix was then removed, and the basal diet was offered ad libitum assuming feed residuals of 10%. The remaining basal diet was removed before the next feeding. After two days for M1 and after seven days for all animals, the feeding of seaweed mix was changed for reasons as described in Section 3. The chemical composition of diet ingredients and of *A. taxiformis* are presented in Table 2. Animals were milked twice daily at 6:00 and 16:00 h, with separate milking units per animal to avoid potential carry-over of milk compounds (like bromoform) between subsequent milkings. Individual milk yield was automatically registered. After sampling, all milk was discarded during the study.

### 2.2. Sample Collection and Measurements

**Figure 1:**
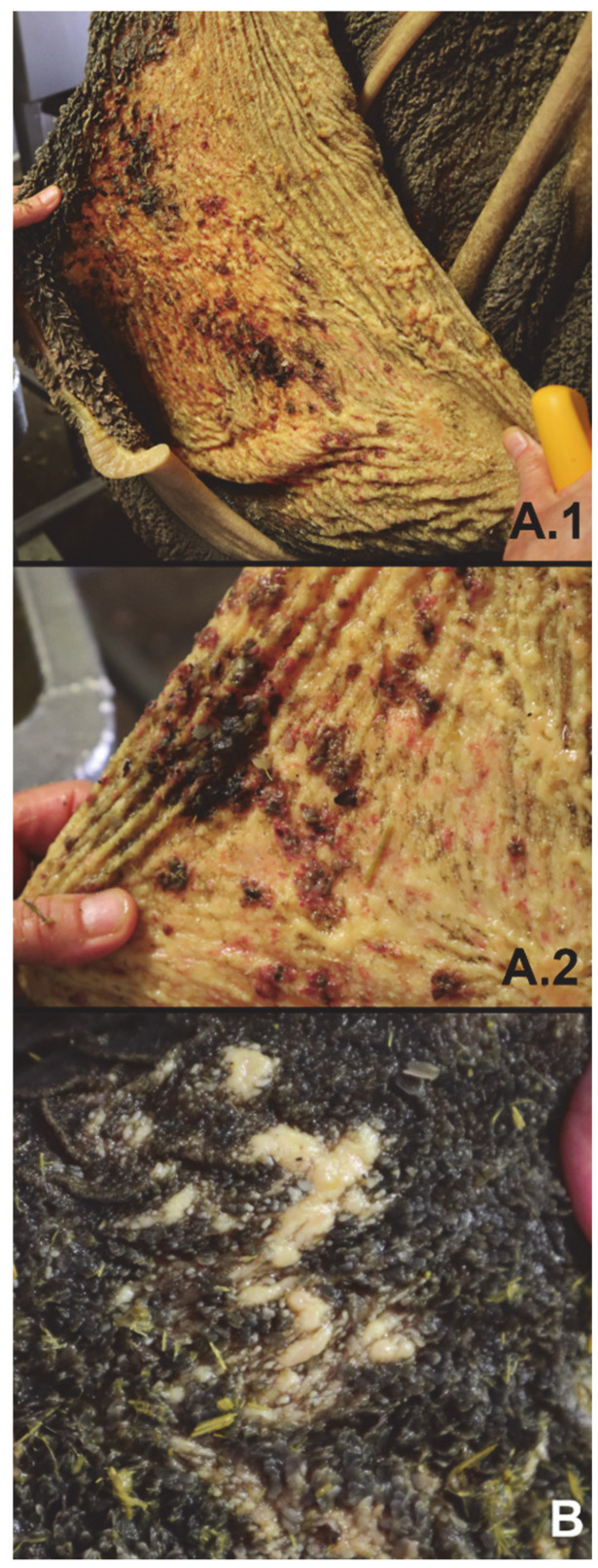
(**A.1**,**A.2**) Overview of rumen wall (**A.1**) and close-up (**A.2**) of cow L1 indicating the absence of rumen papillae on large parts of the rumen wall and presence of hemorrhages and ulcers. (**B**) Close-up of rumen wall of cow L6 with local absence of rumen papillae and thickening of the rumen wall (yellowish white spots).

Feed residues were recorded twice daily before the following feeding event. A sample (ca 200 g) of each feed ingredient (excluding *A. taxiformis*) was collected weekly and stored at −20 °C pending chemical analysis. Samples were analyzed by Eurofins Agro (Wageningen, the Netherlands) for DM (drying at 103 °C), ash (NEN-ISO 5984), crude protein (similar to NEN-ISO 5983-2), crude fat (similar to NEN-ISO 6492), crude fiber (similar to NEN-EN-ISO 6865), starch (NEN-EN-ISO 15915, only maize silage), reducing sugar (NEN 3571), NDF [16] (including amylase), ADF (NEN-EN-ISO 13906), and ADL (NEN-EN-ISO 13906). A sample of *A. taxiformis* was collected at the start of the experiment and analyzed by the laboratory of the department of Animal Nutrition, Wageningen University and Research for DM, ash, crude protein, crude fat, crude fiber, and reducing sugar as described by [17]. Weekly milk samples were collected from all animals on Wednesday PM and Thursday AM for the entire period. A milk sample (10 mL) of each milking event was collected in a tube containing sodium azide (5 µL) for preservation, stored no longer than one day at 4 °C, and analyzed by mid-infrared spectroscopy for fat, protein, lactose, and urea content, as well as somatic cell count. For CHBr_3_ analysis, milk samples (100 mL) were collected from all animals on days −2, −1, 1, 2, 3, 26, 27, and 28. Urine samples (100 mL) for CHBr_3_ analysis were collected on days −2, −1, and 28 from all animals by stimulating the perineum of the cow to induce spontaneous urination. Fecal samples (rectal grab samples; 100 g) were collected from all animals on days −2, −1, and 28. Samples taken at the end of the adaptation period were the control samples for each animal. All samples for CHBr_3_ analysis of milk, urine, and fecal samples were taken 1–3 h after morning feeding and stored immediately at −20 °C pending analysis. At the end of the experiment, two animals with a consistently high seaweed mix uptake were sacrificed, and organs were collected before further processing. Animals received the last seaweed mix 24 h before slaughter. Organ samples were divided into two sub-samples: samples for CHBr_3_ analysis were stored in a plastic jar in −20 °C until further analysis, and samples for histological analysis were stored in 4% buffered formaldehyde until further analysis. Samples were taken from the liver, kidney, rumen wall (from areas indicated in Figure 1), hind leg muscle, and kidney fat. Animal carcasses were sent for destruction and did not enter the food chain. Pathological examination on M1 (280 DIM, 757 kg BW, multiparous) was carried out by the Royal GD (GD Animal Health, Deventer, the Netherlands).

### 2.3. Method for Bromoform Analysis

Bromoform was determined in different matrices using headspace analysis with solid phase micro extraction (SPME) interfaced with two-dimensional gas chromatography and with time-of-flight mass spectrometry (GCxGC-TOFMS). The sample preparation was fully automated. Typically, samples of 10 g of urine, 5 g of milk, 1 g of manure, or 0.5 g of *A. taxiformis* were weighed into 20 mL headspace vials. Milk, manure, and *A. taxiformis* were diluted before analysis. Samples were brought to a total volume of 10 mL with ultrapure water after an internal standard (isotopically labelled CHBr_3_) was added. The auto sampler placed the vial in an oven for 20 min at 70 °C, during which the SPME fiber adsorbs volatile components in equilibrium with the headspace. Next, the SPME fiber was desorbed in the hot injector of the GCxGC system, and the analysis was started. Analyte detection took place with mass spectrometry in full scan mode with 500 scans per second. The detection limit for urine was 1 µg/L, milk 5 µg/L, manure 20 µg/kg, and *A. taxiformis* 50 µg/kg. The detection limit for all organ tissue was 20 µg/kg. One series of urine (day 17) had a detection limit of 2 µg/L due to a reduced retrieval of the internal standard.

### 2.4. Data Analysis

Averages of CHBr_3_ concentration per treatment were calculated based only on values exceeding the detection limit. Data on feed intake and milk yield were measured and presented per individual animal on a daily basis. Milk yield and milk composition data was averaged on all animals within one treatment group on days 1, 8, and 15.

## 3. Results

### 3.1. Experimental Development

Due to significant problems with uptake of the seaweed mix, the experiment was stopped prematurely, and the experimental period lasted 22 days. From the start of the experiment, one of the original animals in the low group had a reduced feed intake, and this animal was replaced by cow L1 on day −3. Cow M1 was euthanized on a decision by the veterinarian on day 13, due to abomasal displacement, probably related to low feed intake and low rumen activity. On day 14, cows L2, L4, L5, L7, L8, M2, and H1 no longer received the seaweed mix due to very low voluntarily intake of the seaweed mix and loss of body condition. Cows L1, L3, L6, and H2 continued to receive the seaweed mix from day 14 onwards, and the seaweed mix treatment for cow H2 was stopped at day 16 due to reduced intake. Animals sent to the slaughterhouse (L1 and L6) for organ sampling were selected on consistently high seaweed mix intake and were therefore from the low treatment group. Feed intake and milk yields were recorded for all animals until day 19. An indicative overview of the seaweed mix uptake per animal per day is presented in Supplementary Table S1. Samples taken for CHBr_3_ analysis were changed to days −2, −1, 1, 9, 10, and 17 for milk, and −2, −1, 1, 10, and 17 for urine and feces (all days expressed relative to first day of offering of seaweed mix).

### 3.2. CHBr_3_ Measurements

The CHBr_3_ content in seaweed used in the present study was 1.26 mg/kg DM. All mean values in Table 3 are based on values that are above the detection limit; when no CHBr_3_ above the detection limit was detected for any animal, the detection limit was presented. In all animals, CHBr_3_ levels were below the detection limit on days −2 and −1 in the milk, urine, and feces. On day one, CHBr_3_ levels in the milk for low, medium, and high treatments were, respectively, 9.1 µg/L (five out of eight animals, min 6.2 µg/L, max 15 µg/L), 11 µg/L (two out of two animals, min 10 µg/L, max 12 µg/L), and below the detection limit. On day nine, CHBr_3_ levels in the milk for all animals in the low and medium treatments and for one cow in the high treatment were below the detection limit, whereas CHBr_3_ in the milk of cow H1 was 35 µg/L. On days 10 and 17, no CHBr_3_ was detected in the milk of any of the animals. At day one and 10, CHBr_3_ was measured above the detection limit in the urine for all animals. Bromoform levels in the urine were 10 µg/L (min 2.0 µg/L, max 29 µg/L), 50 µg/L (min 8.7 µg/L, max 91 µg/L), and 114 µg/L (min 79 µg/L, max 149 µg/L) for the low, medium, and high treatments, respectively, on day one. At day 10, CHBr_3_ levels in the urine were 89 µg/L (min 5.8 µg/L, max 267 µg/L), 148 µg/L (min 27 µg/L, max 269 µg/L), and 127 µg/L (min 107 µg/L, max 146 µg/L) for the low, medium, and high treatments, respectively. No CHBr_3_ was detected in the urine of any of the animals on day 17. None of the fecal samples had CHBr_3_ levels above the detection limit of 20 µg/kg. Organ samples collected upon slaughter of cows L1 and L6 did not have detectable (< 20 µg/kg) CHBr_3_ levels.

### 3.3. Pathology and Histology

**Figure 2:**
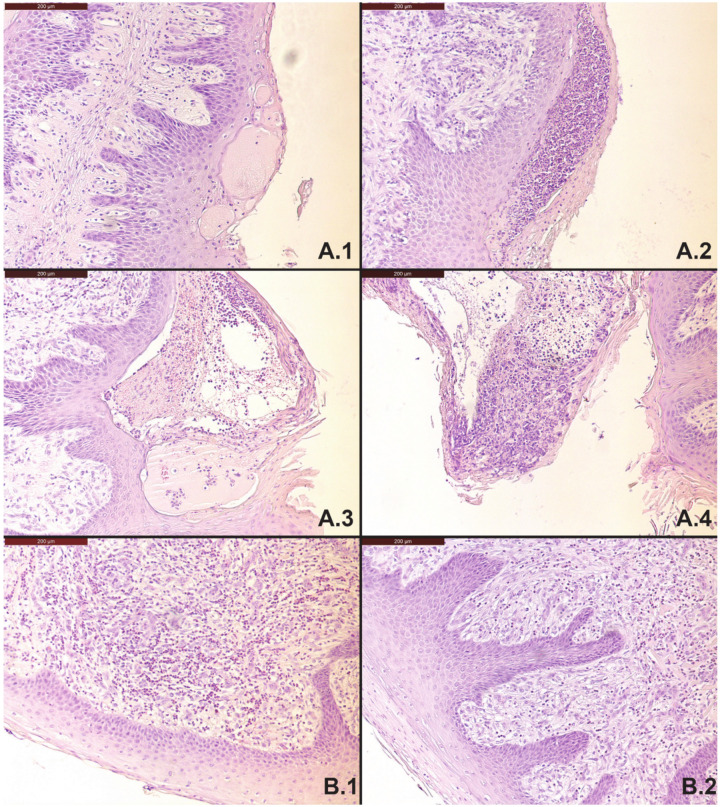
(**A.1**–**A.4**) Histological examination of the rumen wall of cow L1 on parts with a reduced amount of papillae; remaining papillae show formation of exudate filled blisters in the epithelial layer (**A.1**), and invasion of inflammatory cells (polymorphonuclear leukocytes) into these cavities forming micro-abscesses (**A.2**,**A.3**), which leads to ulceration and papillary necrosis (**A.4**). (**B.1**,**B.2**) Histological examination of the rumen wall of cow L6 on local spots with missing rumen papillae and thickening of the rumen wall, and invasion of polymorphonuclear leukocytes (**B.1**), sometimes combined with hyperkeratosis (**B.2**).

The interval between death and post-mortem observations and sampling for both animals was < 1 h. Visual inspection of the rumen wall showed the absence of rumen papillae on a large area, combined with clearly visible hemorrhages and ulcers (cow L1) and local spots with loss of rumen papillae and thickening of the rumen wall (cow L6) (Figure 1). Histological examination of the rumen wall and papillae of cow L1 indicated the formation of blisters in the epithelial layers, aggregation of inflammatory cells that develop into micro-abscesses (pustules), and papillary necrosis in the remaining papillae (Figure 2A.1,A.4). Invasion of inflammatory cells in combination with hyperkeratosis was visible in histological samples of local spots, with loss of rumen papillae and thickening of the rumen wall of cow L6 (Figure 2B.1,B.2). No visual abnormalities were found in the other collected organ samples for both animals.

Pathological examination of cow M1 showed that local pustules with large amounts of bacteria and few hyphae were present in the reticulum. Furthermore, diffusely dispersed inflammation was present in the underlying stroma and muscle layers, consisting of polymorphonuclear leukocytes, combined with gaseous formation. In the serosa, gaseous inflammation was also present.

### 3.4. Feed Intake

Animals regularly refused the seaweed mix (Supplementary Table S1) or distinctively selected against it when mixed with their fresh feed, consequently leading to lower total DMI. Average daily feed intake (DM basis) on days one through six was 0.8 kg (max increase 1.0 kg, max decrease 2.8 kg), 1.6 kg (max increase 0.3 kg, max decrease 3.4 kg), and 2.1 kg (min 0.9 kg, max 3.1 kg) lower for treatments low, medium, and high, respectively, compared to days −6 through −1 (Table 4, excluding cow L1). Several changes in diet composition and feeding strategy were made in order to increase the DMI. On day one, cows H1 and H2 refused the seaweed mix during evening feeding, and both cows received no fresh feed during evening feeding and had subsequently lower feed intake (Table 4). During morning and evening feeding on day two, cows L2, M2, and both cows in the high treatment group refused the seaweed mix, and these cows received new fresh feed after consistent refusal. Cow H2 also refused most of the fresh feed on day two and had a DMI of 6.6 kg/d; no signs of sickness were found. During evening feeding, cow M1 also refused the seaweed mix; this was then mixed with the fresh feed and left overnight, which resulted in complete uptake. On days three to six, no seaweed mix was eaten by cows L2, M2, H1, and H2. Cow M1 again partly refused the seaweed mix on days three to six, which, as before, was mixed with fresh feed during evening feeding. Overall, five out of eight cows in the low treatment group consumed all seaweed mix as planned, while none of the cows in the medium and high treatment groups consumed all seaweed mix. On day seven, four changes in the feeding strategy were made in order to increase the uptake of the seaweed mix. Firstly, the amount of water added to the seaweed mix was raised from 2.0 L to 3.0 L for the low treatment group, 3.5 L for the medium treatment group, and 4.0 L for the high treatment group. Furthermore, a change in diet composition (extra maize and soybean meal, no wheat straw or compound feed, change in grass silage, Table 2) to increase the attractiveness was introduced. Thirdly, the seaweed mix was offered in full during morning feeding, and animals finishing the seaweed mix received fresh feed, whereas for all others, the seaweed mix was mixed with fresh feed after 1–2 h (15 kg fresh feed, approximately 33% DM). Finally, if the seaweed mix in the fresh feed was not consumed before evening feeding, no new feed was offered until the following morning, effectively leading to restricted feeding of those animals. On days seven, eight, and nine, all animals consumed most of the offered feed, but distinctively selected against the seaweed mix. The feeding strategy changed again on day 10. All animals were offered the same seaweed mix as the low treatment group at morning feeding, and if the seaweed mix mixed with the feed was not finished 2 h after morning feeding, all animals received new fresh feed. The seaweed mix treatment was stopped and normal feeding practices were returned to for all animals except cows L1, L3, L6, and H2 from day 14 onwards. Cows L3 and H2 partially consumed the seaweed mix on days 14 and 15, and treatment was stopped for cow H2 on day 15. Cows L1 and L6 consumed all of the seaweed mix from the start until the premature end on day 22.

### 3.5. Milk Yield and Composition

Average milk yield (Table 5) between days one through six was 2.2 kg/d higher (min 0.2 kg/d, max 3.4 kg/d) for the low treatment group, and 0.9 kg/d (max increase 1.1 kg/d, max decrease 3.0 kg/d) and 0.7 kg/d (max increase 1.4 kg/d, max decrease 2.9 kg/d) lower for animals in the medium and high treatment groups, respectively, compared to days −6 through −1 (excluding cow L1). During restricted feeding on days seven through 10, the average milk yield was 1.8 (min 0.0 kg/d, max 5.5 kg/d), 5.2 (min 4.0 kg/d, max 6.4 kg/d), and 4.7 (min 2.1 kg/d, max 7.2 kg/d) kg/d lower for the low, medium, and high treatments, respectively, compared to days one through six.

Milk fat and milk protein content increased for the medium and high groups on day eight compared to day one (Table 6). Milk lactose decreased on day eight and day 15 for the medium group. Milk fat, protein, and lactose yield decreased for all treatments on days eight and 15, and this decrease was most pronounced for the medium and high treatments. The urea content (mg/dL) for all treatments was higher on day eight compared to day one, and lower on day 15 compared to day one. The somatic cell count (SCC) on day eight was higher compared to day one for the medium treatment due to the high SCC of cow I (456−619 cells/mL).

## 4. Discussion

### 4.1. Bromoform Excretion and Residues

Excretion of CHBr_3_ via the urine in the current study was not detected in the days preceding feeding the seaweed mix (control period), whereas it was already detected on the first day after receiving half a portion of the seaweed mix. Rat and mice fed pure CHBr_3_ also excrete CHBr_3_ via the urine [18]. CHBr_3_ was excreted via the urine by all cows on days one and 10, but large individual variation existed, as shown at day 10 in the low treatment group (Table 3). In all treatments, CHBr_3_ was also found in the milk when fed the seaweed mix, whereas no CHBr_3_ was measured in the milk before feeding the seaweed mix or after day 10 of feeding. This pattern is the opposite of what is expected based on the lipophilic nature and partition coefficient of CHBr_3_ among human blood, urine, milk, and cow milk [19]. The partition coefficient of CHBr_3_ indicates that, at equilibrium, CHBr_3_ favors milk over blood and blood over urine [19]. In the current study, an opposite pattern was shown where the CHBr_3_ concentration was higher in the urine, and the concentration was quickly below the detection limit in the milk. This indicates a quick adaptation of the excretion pathway by the animal, which is opposite to the expected excretion pathway based upon the partition coefficient for milk, blood, and urine. Roque et al. [11] did not find a significant increase of CHBr_3_ levels in milk upon feeding *A. armata*. Unfortunately, Roque et al. [11] did not report the time of sampling, sample handling, and processing for CHBr_3_ analysis, which makes comparison difficult. Furthermore, in the study of Roque et al. [11], CHBr_3_ was already detected in the milk of the control group fed no *A. armata*. In the current study, large individual variation existed in the excretion of CHBr_3_ into the milk and was inconsistent over time. No CHBr_3_ was measured in the milk of the low and medium treatment groups on day nine, and all treatments on day 10 and 17. No CHBr_3_ was measured in the urine in any of the treatments on day 17. Absence of CHBr_3_ in the urine or milk of several animals, particularly at day 17, is in line with the zero or small intake of CHBr_3_ containing seaweed mix. However, some animals still continuously ate the seaweed mix during the period when no CHBr_3_ was measured in the urine or milk. In rats, CHBr_3_ can be metabolized into carbon monoxide (CO) and increase blood CO levels [12,20]. During this metabolism, CHBr_3_ reacts with cytochrome p450 (superfamily of enzymes) into an intermediary dihalocarbonyl compound from which different pathways exists, resulting in the formation of CO [21]. Even though CO is toxic for the animal, it is believed that this pathway serves as a detoxification pathway [22]. For chloroform, a trihalomethane like CHBr_3_, phosgene may be formed as an intermediary dihalocarbonyl [21]. For CHBr_3_, it is unknown which intermediary dihalocarbonyl complexes may occur. The authors speculate that an adaptation of the CHBr_3_ metabolism occurs over time in animals consistently receiving CHBr_3_, resulting in several bromide (Br)-containing metabolites not measured in the current study. This is supported by the findings that CHBr_3_ concentrations quickly decrease to levels below detectable levels in the urine and milk during the study, even when several animals continuously consumed the seaweed mix in full. The rumen microbiome might also have an effect on the metabolism of CHBr_3_ into secondary components due to its diversity and complexity, affecting the uptake by the animal. The potential adaptation of the excretion pathway and newly formed Br metabolites warrants further studies investigating the metabolism of CHBr_3_. In future studies, levels of CO and CO_2_, dihalocarbonyl intermediates, and inorganic Br may be evaluated in blood and milk.

Bromoform was not detected in any of the animal tissues, which is in line with other studies where *A. taxiformis* was included in the diet of ruminants [9,10]. Bromoform may be rapidly eliminated from animal tissue in rats, with levels already halved 30 min compared to 15 min after administration, and not detectable anymore after 1–4 h [23]. In the present study, the time of slaughter was 24 h after the last supply of seaweed mix, which may explain why CHBr_3_ was not detected in animal tissues.

Concluding from the current and previous studies, CHBr_3_ does not seem to accumulate in animal tissue, but can be excreted in urine and milk. The exact kinetics of CHBr_3_ excretion, formation of metabolites, and their potential toxicity in ruminants is unknown, and should be further investigated, including the effects on animal wellbeing and the fate of CHBr_3_ and its metabolites in food and manure in short- and long-term studies.

### 4.2. Effect on the Rumen Wall

The rumen wall of both sacrificed animals (low: both animals consistently consumed all offered seaweed) showed loss or even absence of rumen papillae on parts of the rumen wall, with further animal specific abnormalities upon gross examination. Upon histological examination of the rumen papillae, a similar invasion of inflammatory cells was visible in both animals. Aggregation of inflammatory cells is not directly linked to the supplementation of *A. taxiformis* containing CHBr_3_, but is a general immune response to inflammation. Similar abnormalities on visual and histological inspection were found in the euthanized cow I, although the location of this finding was in the reticulum. In a study with sheep fed *A. taxiformis*, comparable histopathological changes were found on the rumen wall and papillae in five out of 10 animals that consumed *A. taxiformis*, with no abnormalities and damage in two out of two control animals not consuming *A. taxiformis* [10]. It is possible that the findings of rumen wall abnormalities, also shown in the study of Li et al. [10], were not related to the consumption of seaweed. Further studies in a well-controlled setting are required to evaluate if the feeding of *A. taxiformis* may have a detrimental effect on rumen wall characteristics.

### 4.3. Feed Intake and Refusal

In the current study DMI reduced numerically by 5.4% (low, excluding cow L1), 8.8% (medium), and 13.5% (high) on days one to six compared to days −1 to −6. The average inclusion of *A. taxiformis* on days one to six was 0.44, 0.82, and 2.48% of feed DM, or 0.24, 0.44, and 1.34% of feed OM for low, medium, and high treatments, respectively. The reduction in DMI at an inclusion level of 0.44% OM is comparable to the 10.8% DMI reduction in the dairy cow study of Roque et al. [11], with inclusion of *A. armata* at 0.5% OM. The same dairy cow study found much larger reductions in DMI (38.0%) at 1.0% OM compared to the 1.34% OM in the current study. The levels of CHBr_3_ in the seaweeds of both studies were comparable: 1.26 mg/kg DM (current study) and 1.32 mg/g DM [11]. Given that, in the present study, animals frequently did not consume the seaweed mix or selected against the seaweed mix, the actual intake of *A. taxiformis* may have been lower than the inclusion levels mentioned previously. This may have resulted in less pronounced declines in DMI. In a dose response study with dairy cows [24], a 22.0% reduction in DMI was shown with the inclusion of *A. taxiformis* at 0.75% of feed DM, which is much higher than the reduction in DMI found at the 0.82% DM *A. taxiformis* inclusion rate in the current study. No effects were visible at the inclusion of 0.25 and 0.50% DM [24]. However, in the study of Stefenoni et al. [24], the average DMI of the control group was already 3–5 kg/d lower when compared to the 0.25 and 0.50% DM groups. In a trial with sheep, the animals regularly refused *A. taxiformis* (1.75 mg/g DM CHBr_3_, as mentioned by Kinley et al. [9] when offered at 2 or 3% of feed OM [10]). In a recent study with beef steers, no reductions in DMI were found when *A. taxiformis* (6.55 mg/g DM CHBr_3_) was fed at inclusion rates of 0.05, 0.10, and 0.20% of feed OM. The current study adopted separate offerings of the seaweed mix twice daily before mixing it through the feed. This may have led to the occurrence of instantaneously high concentrations of CHBr_3_ in the rumen that would not have occurred when seaweed would be completely mixed in the diet, and may have a negative impact on the feed intake, rumen function, and performance of the animals. Overall, the inclusion of ≥ 0.5% OM *Asparagopsis* spp. is likely to reduce the DMI. There is insufficient data to quantify how the inclusion levels of the seaweed or concentration of CHBr_3_ affect feed intake.

### 4.4. Milk Yield and Composition

During days one to six, the average milk yield increased by 15.3% for the low treatment group (excluding cow L1) and lowered by 5.4% and 3.9% for the medium and high treatment groups, respectively, compared to days −6 to −1. A reduction in milk yield when fed *Asparagopsis* spp. has also been observed in other studies. Roque et al. [11] reported a 11.6% milk yield reduction at 1.0% OM inclusion, and Stefenoni et al. [24] a 15.2% reduction at 0.75% DM inclusion. Feed intake is the main driver for milk yield [25], and lower feed intake can explain lower milk yield. The smaller reduction in milk yield in the current study on days one to six compared to other studies can partially be explained by a less pronounced reduction in DMI due to the irregular uptake of the seaweed mix by several cows. Milk composition did not show similar patterns compared to Roque et al. [11], where no changes were found at 0.5% OM and reduced milk protein (%) at 1.0% OM inclusion. The increase in milk fat (%) on day eight compared to day one for the medium and high treatment groups might indicate a transfer of body fat reserves towards the milk. Furthermore, on day eight, all animals were fed restrictively in order to increase the seaweed mix intake, which consequently led to low DMI. The low DMI relative to the milk production level may have led to a negative energy balance, which results in the mobilization of body reserves, mainly in the form of fat comparable to the situation of dairy cows in early lactation [26]. The changes in milk urea (%) on day 15 might be explained by the diet change on day 10, due to the relation with the amount of protein and energy in the diet.

### 4.5. Concerns for Human Consumption

The European Union (EU) and the United States Environmental Protection Agency (EPA) established a maximum level of 100 µg/L and 80 µg/L, respectively, for the sum of the trihalomethanes (chloroform, bromoform, dibromochloromethane, and bromodichloromethane) in drinking water [27,28]. In the current study, the individual CHBr_3_ levels in the milk ranged from 6 to 35 µg/L across all treatments. Thus, the measured CHBr_3_ in milk already contributed 35–44% when compared to the maximum level for drinking water set by the EU or EPA, respectively. *A. taxiformis* is known to contain a wide range of volatile halogen compounds, including other trihalomethanes, dibromochloromethane and bromodichloromethane [29]. To the best of our knowledge, no maximum limits for trihalomethanes are set for animal products like milk. The authors recommend the investigation of such limits for animal products if the use of *A. taxiformis* to reduce enteric methane emissions is approved by national and international regulatory bodies.

## 5. Conclusions

Feeding *A. taxiformis* to lactating dairy cows numerically reduces voluntary feed intake at all treatment levels. Within the confines of the present experiment, CHBr_3_ does not accumulate in lactating dairy cow tissue, but can be excreted in urine and milk when fed *A. taxiformis* containing 1.26 mg/kg DM of CHBr_3_. Abnormalities of rumen wall papillae were identified in cows fed *A. taxiformis*. Further work is required to define long-term effects of *A. taxiformis* on the rumen wall and on the presence and metabolism of CHBr_3_ in milk and urine.

## Figures and Tables

**Table 1:** Amount and composition of the seaweed mix fed daily to dairy cattle at low, medium, or high levels.

Ingredients	Low	Medium	High
*A. taxiformis* (g DM)	67	133	333
Dextrose (g DM)	67	133	333
Wheat (g as fed basis)	500	500	500
Dehydrated beet pulp (g as fed basis)	500	500	500
Water (liters)	2	2	2

**Table 2:** Chemical composition (g/kg DM, unless stated otherwise) of the different ingredients in the basal diet and of the seaweed *A. taxiformis* fed to dairy cows. Diet consisted of grass silage, maize silage, soybean meal, compound feed, and wheat straw, with a ratio of 57:25:12:3:2 ^1^.

Nutrients	Grass Silage ^2^	Maize Silage	Soybean Meal	Compound Feed ^3^	Wheat Straw	*A. taxiformis*
DM (g/kg)	466	348	874	878	886	951
Ash	121	47	70	45	67	510
Crude Protein	203	69	513	115	36	146 ^4^
Crude Fat	43	30	13	10	11	7.5
Crude Fiber	219	181	46	100	404	65
NDF	430	377	242	257	757	ND
ADF	237	209	142	123	454	ND
ADL	14	14	4	8	51	ND
Reducing Sugar	95	12	117	128	30	11
Starch	ND	323	ND	ND	ND	ND
CHBr_3_ (mg/kg DM)	ND	ND	ND	ND	ND	1.26

^1^ Day 10, diet consisted of grass silage, maize silage, and soybean meal only with a ratio of 33:53:14; DM basis. ^2^ Day 10, grass silage was changed to DM = 549 g/kg, ash = 109 g/kg DM, crude protein = 141 g/kg DM, crude fat = 33 g/kg DM, crude fiber = 227 g/kg DM, NDF = 452 g/kg DM, ADF = 244 g/kg DM, ADL = 27 g/kg DM, and reducing sugar = 157 g/kg DM. ^3^ 60% beet pulp, 40% wheat; DM basis. ^4^ Crude protein for all ingredients calculated as N × 6.25. The factor 6.25 may overestimate the crude protein content of macroalgae in general [15]. ND = Not determined.

**Table 3:** Levels of bromoform (CHBr_3_) in milk, urine, and feces of the dairy cows on different days relative to the first day of offering seaweed mix containing *A. taxiformis*. Averages are calculated based on values above the detection limit, otherwise, the detection limit is given.

		Treatments		
	Day	Low	Medium	High
CHBr_3_ Milk	−2	<5	< 5	<5
(µg/L)	−1	<5	<5	<5
	1	9.1 ^1^	11	<5
	9	<5	<5	35 ^2^
	10	<5	<5	<5
	17	<5 ^3^	<5^3^	<5 ^3^
CHBr_3_ Urine	−2	<1	<1	<1
(µg/L)	−1	<1	<1	<1
	1	10	50	114
	10	89 ^4^	148	127
	17	<2 ^3,5^	<2 ^3,5^	<2 ^3,5^
CHBr_3_ Feces	−2	<20	<20	<20
(µg/kg)	−1	<20	<20	<20
	1	<20	<20	<20
	10	<20	<20	<20
	17	<20	<20	<20

^1^ Value based on five out of eight animals. ^2^ Only the milk of cow H1 was above the detection limit. ^3^ None of the values were above the detection limit, even for animals still receiving seaweed. ^4^ Values ranged from 6–267 µg/L. ^5^ Due to reduced retrieval of the internal standard, the detection limit was 2 µg/L.

**Table 4:** Individual dry matter intake (kg/d) during the experiment, and parity and days in milk (DIM) of the dairy cows at the start of the experiment (day −7 relative to the first day of offering the seaweed mix containing *A. taxiformis*).

	Treatments											
	Low								Medium		High	
Cow	L1	L2	L3	L4	L5	L6	L7	L8	M1	M2	H1	H2
Parity	2	1	1	1	1	5	1	1	4	4	2	1
DIM	281	577	538	326	344	521	159	126	280	348	281	282
Day												
−7	^1^	12.3	12.2	13.5	13.2	8.4	10.2	10.1	14.1	13.7	12.9	11.3
−6	^1^	15.8	16.4	19.2	15.8	18.1	16.2	13.1	20.5	18.6	17.7	15.4
−5	^1^	13.2	16.2	18.2	14.5	17.8	15.3	10.1	19.2	18.1	15.3	14.6
−4	^1^	16.1	15.7	16.2	15.5	19.6	17.9	11.4	15.6	16.6	15.8	16.0
−3	^1^	14.9	16.9	17.4	15.3	18.5	15.0	14.4	17.8	17.0	15.3	14.4
−2	18.0	14.2	15.5	18.0	13.4	19.8	16.6	14.3	17.4	18.4	15.3	16.6
−1	17.2	15.3	14.7	17.7	8.9	18.2	14.2	14.3	18.4	15.8	15.5	13.8
1	16.8	16.3	16.3	16.8	9.3	19.8	15.7	14.7	15.9	17.6	10.3	5.4
2	17.0	13.5	14.5	16.3	13.8	20.1	15.0	13.2	13.6	15.9	15.2	6.6
3	14.6	12.9	14.0	12.8	12.1	16.1	12.8	12.0	11.2	16.1	14.3	11.3
4	18.5	15.4	16.3	13.2	14.5	20.7	13.8	11.5	15.9	19.0	16.5	14.0
5	15.5	16.3	16.6	15.6	9.9	19.3	11.4	12.8	15.8	17.6	17.5	15.4
6	20.3	15.7	14.0	16.7	17.7	22.0	9.5	13.2	16.1	20.0	15.5	18.5
7 ^2^	9.9	9.9	9.9	10.0	9.9	10.0	5.0	5.0	4.8	4.9	3.6	3.1
8 ^2^	14.7	9.8	9.9	4.0	9.8	14.8	4.9	14.3	2.1	3.0	3.1	1.2
9 ^2^	14.3	7.0	11.0	10.2	13.8	19.5	7.6	7.7	2.8	8.6	7.7	5.3
10 ^2^	9.6	9.8	9.8	9.8	9.6	19.6	9.8	9.6	8.1	9.8	9.7	9.8
11	13.6	13.3	14.9	12.0	14.6	14.8	14.5	14.7	8.8	14.9	8.9	11.4
12	14.2	14.6	14.4	14.8	14.7	14.8	14.7	14.8	5.6	14.8	13.4	9.9
13	4.7	14.6	14.0	14.7	14.6	9.7	4.7	4.8	^3^	14.7	14.7	^4^
14	17.4	18.8	18.7	19.3	19.0	19.3	17.8	18.5	^3^	19.7	18.1	11.2
15	17.1	16.2	17.4	18.8	17.6	19.6	18.5	18.5	^3^	16.9	16.6	13.0
16	23.9	17.2	17.7	24.1	18.0	27.8	23.0	21.9	^3^	19.7	16.7	22.4
17	19.8	16.3	19.3	21.9	20.4	20.5	18.7	22.1	^3^	20.2	17.0	17.5
18	16.5	16.3	16.3	20.7	16.1	21.0	15.8	16.2	^3^	20.6	15.7	17.6
19	19.3	17.5	19.0	19.6	19.4	24.7	16.6	19.2	^3^	19.1	18.7	16.7

^1^ No data available due to the entrance of the animal into experiment on day −3. ^2^ Animals were fed restrictedly on these days. ^3^ No data available due to euthanasia of the animal. ^4^ No data available due to no registration of feed intake.

**Table 5:** Individual milk yield (kg/d) of the dairy cows during the experiment. Day −6 is relative to the first day of offering the seaweed mix containing *A. taxiformis*.

	Treatments											
	Low								Medium		High	
Cow	L1	L2	L3	L4	L5	L6	L7	L8	M1	M2	H1	H2
Day												
−6	^1^	11.7	10.4	18.3	10.0	11.8	7.8	7.6	22.9	12.7	15.3	19.9
−5	^1^	12.1	10.7	16.7	16.4	16.6	19.9	13.2	22.5	13.1	14.7	18.3
−4	^1^	11.6	9.9	15.9	15.1	13.0	21.1	13.4	23.8	11.7	16.8	17.4
−3	^1^	14.0	9.6	14.8	19.3	14.7	21.0	13.5	23.6	11.7	18.8	19.7
−2	7.0^5^	13.7	10.5	14.6	17.1	14.9	21.3	8.9 ^5^	23.4	11.7	19.5	20.2
−1	22.6	14.5	8.5	16.6	17.0	16.0	22.2	16.3	20.4	11.8	18.4	19.5
1	22.9	13.3	8.6	17.5	16.4	15.2	21.4	16.0	22.0	11.6	17.1	18.9
2	23.6	13.2	9.2	15.7	16.6	15.0	21.8	15.6	21.8	11.5	13.1	13.6
3	23.1	12.9	13.9	18.9	18.7	15.3	21.4	15.8	20.9	13.6	17.8	13.1
4	22.4	16.3	14.9	20.8	20.6	13.5	20.3	16.2	19.6	14.5	21.8	16.4
5	22.8	14.6	14.8	21.5	19.5	15.0	19.6	16.4	18.5	12.8	22.5	17.8
6	22.2	15.6	15.0	20.4	20.1	14.4	19.3	13.2	15.8	15.5	19.8	18.0
7 ^2^	23.1	16.4	14.2	23.8	20.9	14.4	16.4	15.5	17.3	14.5	18.3	19.1
8 ^2^	21.6	13.4	12.4	14.3	19.0	12.0	14.8	13.3	15.2	9.4	10.5	15.4
9 ^2^	21.2	13.7	10.1	13.0	17.1	13.1	13.9	15.6	11.3	7.7	9.7	10.4
10 ^2^	19.6	8.5	7.7	15.3	17.6	12.4	15.5	15.5	9.6	5.6	7.3	12.0
11	18.3	10.1	13.4	6.4	15.9	15.0	15.9	15.1	11.5	6.8	10.1	13.8
12	18.6	11.7	12.4	9.4	16.2	11.4	16.3	14.8	9.5	9.0	4.3 ^5^	14.3
13	18.4	12.1	12.7	19.7	16.6	13.1	17.5	15.7	^4^	6.8	10.6	14.3
14	22.2	13.6	14.6	16.4	20.7	9.1	18.4	17.0	^4^	9.0	14.0	13.6
15	21.2	14.0	14.6	18.4	18.4	3.4 ^5^	14.8	16.4	^4^	9.9	7.1 ^5^	14.1
16	^3^	^3^	14.6	20.0	20.7	3.9 ^5^	18.4	16.7	^4^	10.3	^3^	14.8
17	9.4 ^5^	8.9 ^5^	15.8	21.5	20.3	7.1	19.7	15.9	^4^	10.5	8.4 ^5^	16.0
18	22.3	14.2	14.9	21.0	20.4	14.2	20.6	16.1	^4^	12.0	17.2	16.6
19	21.6	16.5	13.6	22.8	21.3	8.1	20.6	16.3	^4^	13.1	18.8	16.3

^1^ No data available due to the entrance of the animal into the experiment on day −3. ^2^ Animals were fed restrictedly on these days. ^3^ No data available due to system error. ^4^ No data available due to euthanasia of the animal. ^5^ Data based on one milking event.

**Table 6:** Milk production and composition of dairy cows at several days (relative to the first day of offering the seaweed mix containing *A. taxiformis*) during the experimental period.

		Treatments		
	Day	Low	Medium ^1^	High
Yield (gram/day)				
Fat	1	806	788	771
	8	746	752	730
	15	733	595	485
Protein	1	652	695	650
	8	608	521	493
	15	595	436	385
Lactose	1	738	751	838
	8	671	504	564
	15	677	379	474
Milk composition (%)				
Fat	1	4.91	4.69	4.28
	8	4.94	6.12	5.64
	15	4.84	6.01	4.57
Protein	1	3.97	4.14	3.61
	8	4.02	4.23	3.81
	15	3.93	4.40	3.63
Lactose	1	4.49	4.47	4.65
	8	4.45	4.10	4.35
	15	4.47	3.83	4.47
Urea (mg/dL)	1	17.6	18.5	19.6
	8	23.1	22.9	23.0
	15	14.2	13.3	14.6
SCC (cells/mL)	1	56,709	64,841	36,325
	8	66,923	276,219	50,757
	15	65,742	170,338	63,600

SCC = Somatic cell count. ^1^ All values on day 15 for the medium treatment group are only based on cow J.

## Data Availability

Data is contained within the article or Supplementary Materials.

## Supplementary Materials

The following are available online at https://www.mdpi.com/2304-8158/10/3/584/s1, Table S1: Indication on the intake of the seaweed mix containing *Asparagopsis taxiformis* per animal per day since the first offering of the seaweed mix on day one.
