# Supplementary material for: Safety and Transfer Study: Transfer of Bromoform Present in Asparagopsis taxiformis to Milk and Urine of Lactating Dairy Cows
Source: Foods. 2021 Mar 10;10(3):584. doi: 10.3390/foods10030584 (PMC7998480; doi:10.3390/foods10030584)
Supplement: Supplementary file 1 [file foods-10-00584-s001.pdf]

foods

MDPI

Supplementary Table S1. Indication on the intake of the seaweed-mix containing Asparagopsis taxiformis per animal per day since first offering of the seaweed-mix on day 1.

	Treatments											
	Low								Medium		High	
Cow	L1	L2	L3	L4	L5	L6	L7	L8	M1	M2	H1	H2
Day												
1	+	+	+	+	+	+	+	+	+	+	+/-	+/-
2	+	-	+	+	+	+	+	+	+/-	-	-	-
3	+	-	+	+	+	+	+	+	+/-	-	-	-
4	+	-	+	+/-	+	+	+	+/-	+/-	-	-	-
5	+	-	+	+/-	+	+	+	+/-	+/-	-	-	-
6	+	-	+	+/-	+	+	+	+/-	+/-	-	-	-
7[1]	+	+/-	+	+/-	+/-	+	+/-	+/-	+/-	+/-	+/-	+/-
8[1]	+	+/-	+	+/-	+/-	+	+/-	+/-	+/-	+/-	+/-	+/-
9[1]	+	+/-	+/-	+/-	+/-	+	+/-	+/-	+/-	+/-	+/-	+/-
10[1]	+	+/-	+	+/-	+/-	+	+/-	+/-	+/-[2]	+/-[2]	+/-[2]	+/-[2]
11	+	+/-	+	+/-	+/-	+	+/-	+/-	+/-[2]	+/-[2]	+/-[2]	+/-[2]
12	+	+/-	+	+/-	+/-	+	+/-	+/-	+/-[2]	+/-[2]	+/-[2]	+/-[2]
13	+	+/-	+	+/-	+/-	+	+/-	+/-	[3]	+/-[2]	+/-[2]	+/-[2]
14	+	NS	+/-	NS	NS	+	NS	NS	[3]	NS	NS	+/-[2]
15	+	NS	+/-	NS	NS	+	NS	NS	[3]	NS	NS	+/-[2]
16	+	NS	+/-	NS	NS	+	NS	NS	[3]	NS	NS	NS
17	+	NS	+/-	NS	NS	+	NS	NS	[3]	NS	NS	NS
18	+	NS	+	NS	NS	+	NS	NS	[3]	NS	NS	NS
19	+	NS	+	NS	NS	+	NS	NS	[3]	NS	NS	NS
20	+		+/-			+						
21	+					+						
22	+					+						

NS = No seaweed-mix offered.
+ = Ate all seaweed-mix
+/- = Partially ate seaweed-mix
- = Did not ate seaweed-mix
[1]Animals were fed restrictedly on these days.
[2]Animals were fed the same dose as the Low treatment, 67g dry matter of *A. taxiformis*.
[3]No data available due to euthanasia of animal I.
